# Ibuprofen reduces inflammation, necroptosis and protects photoreceptors from light-induced retinal degeneration

**DOI:** 10.1186/s12974-024-03329-8

**Published:** 2025-01-28

**Authors:** Ping-Wu Zhang, Zi-He Wan, Weifeng Li, Abhishek Vats, Kunal Mehta, Laura Fan, Lingli Zhou, Sean Li, Gloria Li, Casey J. Keuthan, Cynthia Berlinicke, Cheng Qian, Noriko Esumi, Elia J Duh, Donald J. Zack

**Affiliations:** 1 Department of Ophthalmology, Wilmer Eye Institute, Johns Hopkins University School of Medicine, Baltimore, MD 21231 USA; 2 Department of Genetic Medicine, Johns Hopkins University School of Medicine, Baltimore, MD 21231 USA; 3 Department of Neuroscience, Johns Hopkins University School of Medicine, Baltimore, MD 21231 USA; 4 Department of Molecular Biology and Genetics, Johns Hopkins University School of Medicine, Baltimore, MD 21231 USA

**Keywords:** Ibuprofen, Retina, Photoreceptor, Retinal degeneration, Retinitis pigmentosa, Macular degeneration, Necroptosis, Inflammation, Neuroprotection

## Abstract

**Background:**

The retinal degenerative diseases retinitis pigmentosa (RP) and atrophic age- related macular degeneration (AMD) are characterized by vision loss from photoreceptor (PR) degeneration. Unfortunately, current treatments for these diseases are limited at best. Genetic and other preclinical evidence suggest a relationship between retinal degeneration and inflammation. To further explore this relationship, we tested whether Ibuprofen (IBU), an FDA-approved non-steroidal anti-inflammatory drug (NSAID), could promote PR survival and function in a mouse model of light damage (LD)-induced PR degeneration.

**Methods:**

LD was induced by exposing mice to 4000 lx of light for 2–4 hours (h). IBU (100 or 200 mg/kg) or vehicle was administered by daily intraperitoneal injection. Retinal structure and function were evaluated by spectral-domain optical coherence tomography (SD-OCT) and electroretinography (ERG). Cell death genes were analyzed at 24 and 72 h after LD using the Mouse Pan-Cell Death Pathway PCR Array (88 genes). The cellular location and protein expression of key necroptosis genes were assessed by immunohistochemistry.

**Results:**

Retinal outer nuclear layer (ONL) thickness in vehicle-injected LD animals was 8.7 ± 0.6% of retinas without LD (*p* < 0.0001). In IBU 200 mg/kg treated mice, central ONL thickness was 74.9 ± 7.7% of untreated retinas (*p* < 0.001). A-wave and b-wave ERG amplitudes were significantly preserved in IBU-treated animals. IBU significantly inhibited retinal inflammation. Twenty-four hour after LD, retinal mRNA expression for the inflammatory-factors tumor necrosis factor (*Tnf*), interleukin-1 beta (*Il1B*), and C-C motif chemokine ligand 2 (*Ccl2*) increased by 10-, 17-, and 533-fold, respectively; in IBU-treated animals, the expression levels of these inflammatory factors were not significantly different from no-LD controls. Expression of key necroptosis genes, including *Ripk3* and *Mlkl*, were upregulated in LD vehicle-treated mice, but dramatically reduced to near no LD levels in LD IBU-treated mice. Microglia activation and MLKL protein upregulation were observed primarily in photoreceptors 12 h after LD, as assessed by immunohistochemistry. IBU reduced the upregulation of MLKL protein and microglia migration in the ONL and outer plexiform layer (OPL) of treated retinas.

**Conclusions:**

Systemic administration of the anti-inflammatory drug IBU partially protected mouse retinas from light-induced photochemical damage and inhibited both inflammation and the necroptosis cell death pathways. Our results suggest that NSAIDs may provide a promising therapeutic approach for treatment of the human retinal degenerative diseases.

**Supplementary Information:**

The online version contains supplementary material available at 10.1186/s12974-024-03329-8.

## Background

Retinal photoreceptor (PR) cells are the neurons that sense light and generate an electrical signal through phototransduction, and this signal, which gets further processed and propagated to other retinal cell types, is ultimately transmitted to the brain by the optic nerve for visual perception. A variety of diseases can cause damage and degeneration of PR cells, including the common disease age-related macular degeneration (AMD) and the orphan disease retinitis pigmentosa (RP). AMD, which is the largest cause of irreversible blindness in elderly adults, is a polygenic disease with multiple genetic and environmental risk factors [1]. Although the neovascular or wet form of the disease has effective treatments available, the more common atrophic, or dry form of the disease is only minimally treatable, with the first FDA treatment only being approved in 2023 [2, 3]. RP is genetically and clinically heterogeneous, and treatment options are limited [4–6]. There is thus a need to develop improved therapeutic modalities for the retinal degenerative diseases.

Although pegcetacoplan (Syfovre) and avacincaptad pegol (Izervay), both complement mediators, were recently approved for the treatment of atrophic AMD, these drugs require recurring (monthly or bimonthly) ocular injection, not ideal for patients, and their efficacy remains a subject of debate [7, 8]. Active efforts to develop gene therapy and cell replacement-based therapies for the retinal degenerations are also making progress [9]. In 2023, the FDA approved voretigene neparvovec (Luxturna), a treatment for RP patients that have mutations in *RPE65* (~ 1–2% of RP cases), that introduces a normal copy of *RPE65* directly into retinal cells [10]. Another, perhaps complementary approach, which could be applied to all forms of retinal degeneration, is the development of neuroprotective strategies designed to directly promote the survival and function of remaining PRs [11, 12].

Anti-inflammatory drugs are promising options for neuroprotective treatments since inflammation has been implicated as a prominent feature of both AMD and RP [13, 14]. However, there have been mixed results regarding the use of anti-inflammatory drugs in preclinical and clinical studies for treating retinal degeneration [15, 16]. A previous study indicated that the anti-inflammatory potential of the steroidal drug dexamethasone had neuroprotective activity against a light-induced damage rat model [17–19]. However, steroids did not prevent PR degeneration in light-exposed T4R rhodopsin mutant dogs [20]. While steroidal medications, including dexamethasone, progesterone, and progestins, have demonstrated improvement in retinal cell survival in mouse models, extended corticosteroid use has been shown to cause serious side effects [16, 17]. In mouse studies, intravitreal injection of thrombospondin-1 has been reported to protect against light-induced retinal degeneration via dual anti-inflammatory and anti-angiogenic functions [21]. In a human association study (between long-term use of NSAIDs and AMD), nonsteroidal anti-inflammatory drugs (NSAIDs) have been reported to possibly have a protective effect in AMD [22]. Additionally, the NSAID Ibuprofen (IBU), which acts by reducing production of the pro-inflammatory prostaglandins by inhibiting COX-1 and COX-2 [23], has been shown to provide neuroprotection in transgenic mouse models of both Parkinson’s disease and oxygen-induced retinopathy [15, 24].

In an effort to further study the possible role of inflammation in retinal degeneration, and explore the possibility of developing modulation of inflammation as an approach for the treatment of retinal degeneration, we tested the ability of IBU to promote PR survival and function in a mouse model of light-induced PR degeneration [25, 26]. High-intensity light exposure has been reported to be associated with some forms of AMD and RP [27, 28]. Furthermore, an association between sunlight exposure and AMD visual impairment was reported in an Indian fishing community [29]. While there are good mouse models for several forms of RP, there is neither an ideal nor generally accepted animal model for AMD. Although not a specific model for AMD nor RP, light damage (LD) is commonly used as an acute retinal degeneration model that shares some pathology with both RP and AMD [30]. Not only has exposure to high light levels been commonly used to model acute retinal degeneration in rodents, light has also been found to impact disease severity in genetic models of RP. Naash et al. reported that light accelerated PR degeneration in transgenic mice expressing mutant Rhodopsin (P23H) [31]. Moreover, increased environmental light intensity also accelerated cell death in retinal degeneration 10 (rd10) mouse retinas, while mice reared in a dark environment demonstrated a delayed degenerative phenotype [32, 33].

In this study we show that IBU can partially protect PRs against light-induced retinal damage in albino mice, and have explored the death pathways associated with LD that are modulated by IBU treatment.

## Methods

### Animals

Ten- to twenty-week-old female BALB/cJ mice were purchased from Jackson Labs (#000651, USA) and maintained with temperatures of 65–75 °F and 40–60% humidity in a 12-h light/12-h dark cycle, unless otherwise noted. (We also did the LD in male mice as well, and found that by OCT and ERG, IBU had similar PR protection.) All experiments were approved by the Institutional Animal Care and Use Committee (IACUC) of the Johns Hopkins University School of Medicine and in compliance with the Association for Research in Vision and Ophthalmology (ARVO) statement for the ethical use of animals in ophthalmic and vision research.

### Light damage (LD)

Mice were randomly divided into groups (*n* = 4–10 mice per group, depending on the experiment) and dark-adapted for twelve hours before light exposure. Mice used for Fig. 3a and b were 13–20 weeks old and all others were 8–12 weeks old. Mice were exposed to 4000 lx cool-fluorescent light in an eight-compartment box for two to four hours in the afternoon or evening (two hour for mice < 12 weeks old, four hour for mice 13–20 weeks old). Mice were placed back in regular light cycle conditions following LD.

### IBU administration

Ibuprofen sodium salt was purchased from Sigma (I1892-100G, USA) and dissolved in a normal saline solution containing 5% DMSO. 10 to 20-weeks old mice were administered either 200 mg/kg IBU or vehicle by daily intraperitoneal (IP) injection starting five days before LD and for up to five days post-LD (ten IP injections in total), unless otherwise described.

### Spectral-domain optical coherence tomography (SD-OCT)

Non-invasive SD-OCT (Bioptogen, USA) was utilized to assess retina structure. For this procedure, mice were anesthetized by IP injection of 100 mg/mL ketamine hydrochloride (Dechra, USA) and xylazine (10 mg/mL, Dechra, Latvia). Contralateral eyes not undergoing OCT imaging were kept hydrated using a PBS swab and by applying 0.3% hypromellose (Genteal Tears, Alcon, USA) periodically. Mice were positioned with their head toward the projection of the laser for OCT image capture. Six OCT images were averaged to reduce noise, and retinal layers were measured using the caliper function included in the Bioptogen software.

### Electroretinography (ERG)

Scotopic ERG was performed on mice using a Celeris instrument (Diagnosys, USA). All mice were dark-adapted overnight and were anesthetized by the same method described for SD-OCT. Before the test, mice were given 1%Tropicamide Ophthalmic USP solution (Bausch + Lomb, USA) to induce mydriasis. Anesthetized mice were placed on a heating pad and two stainless steel needle electrodes were inserted into the skin near the head/tail of the animal, serving as reference and ground electrodes, respectively. Light-guiding electrodes were brought in contact with the eyes following the application of 0.3% hypromellose (Millipore-Sigma, USA) to the corneal surface. Mice were stimulated with full-field, green light flashes at scotopic intensities of 0.01, 0.1, 1.0, 3.0 and 10.0 cd.s/m^2^, and 5 sweeps were performed for each value. The amplitudes of scotopic a-waves and b-waves were analyzed using Espion software (Diagnosys, USA).

### Tissue histology

Animals were euthanized by CO2 inhalation 15 days after LD. For positioning, eyes were marked with a cautery pen on the superior part of each eye. These marked eyes were immediately removed by exposing the globe using the index finger and thumb and inserting curved forceps behind the globe, pulling the optic nerve outward. Eyes were washed with PBS followed by fixation in 4% PFA (Electron Microscopy Sciences, USA) in PBS. A small incision was made to the cornea to increase exposure of the PFA to the retina. The eyes were fixed for 30 min before removing the cornea and lens. The remaining eyecups were kept in the PFA for an additional 1.5 h, followed by a PBS wash for 10 min, then 30 min of incubations in 5%, 10%, 20%, and 40% gradients of sucrose in PBS. Finally, eyes were soaked in 1:1 40% sucrose and O.C.T. compound mixture (Electron Microscopy Sciences, USA) overnight. Eye cups were embedded in O.C.T. blocks and eyes were oriented by keeping the cautery pen mark on the right-hand corner. The O.C.T. blocks were flash-frozen in a liquid nitrogen bath containing 2-methyl butane for cryopreservation. Eight or twelve microns thick cryosections containing the optic nerve head were sectioned on to super frost glass slides (Sakura Finetek, USA). For histological analysis, a modified H&E staining protocol was used. Briefly, PFA-fixed, frozen sections on slides (FisherScientific, USA) were air-dried for 10 min followed by 10 min of submersions in 1% PFA and in PBS. Slides were sequentially submerged in the following: 95% ethanol for 2 min, hematoxylin for < 1 min, 3x dipping in bluing solution, 3x dipping in tap water, 3x dipping in 70% alcohol where the slides were submerged for 1 min, 2x dipping in eosin, 5x dipping in 95% alcohol submerged for 1 min, 5x dipping in new 95% alcohol submerged for 1 min, 5x dipping in 100% alcohol and kept it for 1 min, 5x dipping in new 100% alcohol and kept it for 1 min, 5x dipping in xylene and kept for 1 min, until finally switching to new xylene where the slides were kept and coverslips were applied with mounting solution (Invitrogen, USA). Slides were dried for 24 h before imaging. Six images per eye were taken at different sections of the retina and the number of nuclei in the outer nuclear layer was manually counted using Image J.

### Immunofluorescence assay (IFA)

Preparation of mouse retinal cryo-sections were similar to H&E histology, except when specified otherwise. For IFA, air-dried slides were blocked with TBST solution (Tris-buffered saline with 0.1% Tween 20 detergent) containing 5% donkey serum (Millipore Sigma, USA) for 2 h and stained overnight at 4 °C with primary antibodies (in blocking solution). Slides were washed three times with TBST at room temperature (5 min each wash) before incubating with secondary antibodies at room temperature for 1 h (in blocking solution). Slides were then washed with TBST one time, for 5 min, then slides were stained with Hoechst 33,342 (1:1000 in PBS, Millipore Sigma, USA) for 5 min. Slides were washed with TBST another 2 times (5 min each). The stained tissue was covered with a coverslip attached by a mounting solution. Primary antibodies and concentrations used in this study were as follows: rabbit anti-MLKL polyclonal antibody (Invitrogen, USA), 1: 200; goat anti-Iba1 polyclonal antibody (Abcam, USA), 1:200; mouse anti-rhodopsin monoclonal antibody (Millipore-Sigma, USA), 1:400; chicken anti-GFAP polyclonal antibody (Invitrogen, USA), 1:200. The following secondary antibodies were used (all at 1:1000) for these studies: donkey anti-goat IgG (Alexa Fluor^®^ 594), donkey anti-mouse IgG (Alexa Fluor^®^ 488), donkey anti-mouse IgG (Alexa Fluor^®^ 488), and donkey anti-chicken IgG (Alexa Fluor^®^ 488).

### RNA extractions

Mice were sacrificed by cervical dislocation at the described endpoints. Retinas were collected 24 h and 72 h post-LD and immediately flash-frozen in liquid nitrogen. Total RNA was extracted using TRIzol (ThermoFisher Scientific, USA) and the Purelink RNA Mini Kit (Invitrogen, USA) with minor modifications, including on-column DNase digestion. Samples were incubated in 500 µL TRIzol for 5 min on ice, then homogenized. After adding 0.2 mL chloroform per mL of TRIzol and vortexing, the mixture was incubated on ice for two to three minutes before centrifuging at 20,000 𝗑 g for 15 min at 4 °C. The clear, aqueous layer was transferred to a new tube, mixed well with an equal volume of 70% ethanol, and transferred to a spin cartridge. Flow-through was discarded after centrifuging at 12,000 𝗑 g for 30 s. The cartridge was washed with 350 µL Wash Buffer I (Purelink RNA Mini Kit) and centrifuged again to remove DNA, then 75 µL DNase (QIAGEN) was added and incubated for 20 min. Another 350 µL Wash Buffer I was added before centrifuging the spin cartridge again, followed by two washes with 500 µL Wash Buffer II (Purelink RNA Mini Kit) with two minutes centrifugation for each wash at 20,000 𝗑 g. Cartridges were rotated 180 degrees and centrifuged again at 20,000 𝗑 g for two minutes before eluting the RNA with 50 µL RNase-free water, incubating for three minutes, and centrifuging at 20,000 𝗑 g for two minutes. The RNA concentration and purity were determined using a NanoDrop spectrophotometer (ThermoFisher Scientific, USA).

### Reverse transcription (RT)

Reverse transcription of purified RNA into cDNA was performed using the iScript cDNA Synthesis kit (BioRad, USA). In an RNase-free 0.2 mL PCR tube, 250 ng of purified retina RNA was combined with 4 µL 5x iScript Reaction Mix (a unique blend of oligo dT and random hexamer primers) and 1 µL of the iScript Reverse Transcriptase (from modified Moloney murine leukemia virus/MMLV) for a total reaction volume of 20 µL (volume brought up with nuclease-free water). Following the manufacturer’s instructions, the priming time was set at 25 °C for five minutes, the reverse transcription phase at 46 °C for 20 min, and enzyme inactivation at 95 °C for one minute.

### Quantitative polymerase chain reaction (qPCR, or real-time PCR)

An iTaq™ Universal SYBR Supermix kit (BioRad, USA) and specific primers (Table S1) were used for qPCR as per the kit protocol. Briefly, the amplification protocol included one minute of polymerase activation at 95 °C, followed by 40 cycles of 10 s denaturation at 95 °C, 30 s annealing and extension together at 60 °C, and a final extension for one minute at 72 °C, with a hold temperature of 8 °C. Fluorescence data was collected at the end of each PCR cycle. Amplicon specificity was verified by melt curve analysis, performed from 65 °C to 95 °C with a gradual increase of 0.5 °C every five seconds. Relative gene expression levels were calculated using the 2^−ΔΔCt^ method, with selected housekeeping genes serving as internal controls (Table S1).

### Cell death pathway screening

The Mouse Pan-Cell Death Pathway PCR Array (Real Time Primers LLC, USA) containing 88 primer sets directed against cell death genes and eight housekeeping gene primer sets were used for qPCR analysis. These 88 target genes span ten different cell death pathways, including apoptosis (intrinsic and extrinsic), autophagy, ferroptosis, necroptosis, pyroptosis, netosis, entosis, methuosis, and parthanatos (Table S2). qPCR was performed in a 2 µL reaction volume using SsoAdvanced™ SYBR Green Master Mix or iTaq™ SYBR Green Master Mix (BioRad, USA) according to manufacturer’s instructions. C_t_ values of target genes were normalized to the geometric mean of the eight housekeeping genes. Student’s t-tests were applied for statistical analysis. Genes with a p-value < 0.1 and a log_2_ fold-change > 0.6 or < -0.6 were considered significantly upregulated or downregulated, respectively.

### Statistical analysis

Differences between two groups were assessed using unpaired Student’s t-tests, and differences between more than two groups were assessed using One-way ANOVA. GraphPad Prism (version 10) was used for graphic figures. Data were presented as mean ± SEM. Statistical significance: *p < 0.05, **p < 0.01, ***p < 0.001 and ****p < 0.0001.

## Results

### IBU preserved retinal morphology following light-induced PR degeneration

**Fig. 1:**
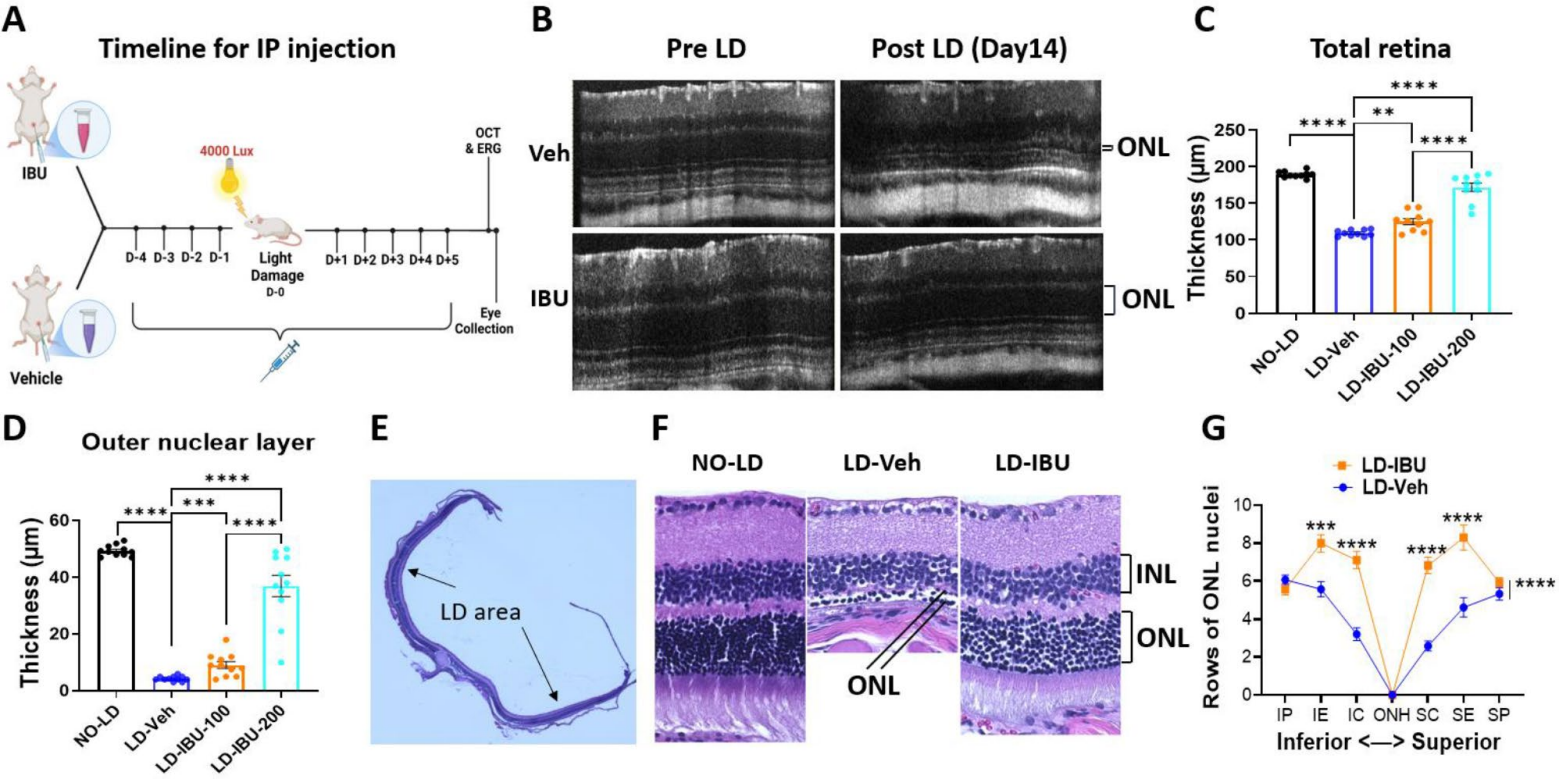
Protective effect of IBU on retinal structure and morphology following LD (Representative retinal layers and position of SD-OCT measurement are shown in Figure S1). **A** Schematic showing timeline for IBU and vehicle IP injections and LD. OCT and ERG were performed 14 days after LD. Eyes were collected for histological analysis 15 days after LD. **B** Representative OCT images showing changes in the retinal structure 14 days after LD, with and without 200 mg/kg IBU treatment. **C-D** Total retina thickness (**C**) and outer retinal layer thickness (**D**) measured from OCT images (as shown in panel B) across different experimental groups: NO-LD (Control), LD-Veh (Vehicle treated with LD), LD-IBU-100 (100 mg/kg IBU-treated with LD), and LD-IBU-200 (200 mg/kg IBU-treated with LD). Dots represent individual mice, *n* = 10 mice per experimental group. **E** Representative image of an H&E-stained retina showing the area affected by LD. **F** Representative images of H&E-stained retinas of a mouse not exposed to LD and LD retinas from mice treated with either vehicle or 200 mg/kg IBU. **G** Rows of ONL nuclei counted from H&E-stained LD retinas (as shown in panel **F**) of mice treated with either IBU or vehicle. Abbreviations: ONL, outer nuclear layer; INL, inner nuclear layer; IP, inferior peripheral; IE, inferior equatorial; IC, inferior central; ONH, optic nerve head; SC, superior central; SE, superior equatorial; SP, superior peripheral

To determine whether IBU could protect PRs from acute light damage, we tested the drug’s neuroprotective effects at two doses (100 mg/kg and 200 mg/kg) and compared the effects to 5% DMSO in saline as a vehicle control (Fig. 1A). Based on previous publication [34] and our pilot experiments characterizing retinal morphology changes following LD, for IBU administration we chose to perform daily systemic IBU injections for the five days before LD, followed by daily IBU injection for the five days after light exposure. As expected, vehicle-treated mice exposed to LD had severe PR loss, with the retinal outer nuclear layer (ONL) thickness, as assessed by OCT, decreased to 4.3 ± 0.6 μm (8.7 ± 0.6% of the no LD ONL which was 49.2 ± 1.2 μm) when measured 14 days post-LD (average of both eyes of each animal). In contrast, IBU injection significantly preserved ONL thickness in the central region of the mouse retina at 37 ± 7.7 μm (74.9 ± 7.7% of the ONL with no LD; *p* < 0.001) for the 200 mg/kg dose. Preservation of retinal morphology was mild with 100 mg/kg treatment, but was highly significant with 200 mg/kg treatment (Fig. 1B-D, Figure S1-2).

We confirmed IBU-mediated protection of retinal structure by histology 14 days after LD. H&E staining showed that there was preservation of both total retina thickness and in terms of the number of rows of ONL nuclei in IBU-treated LD mice (Fig. 1E-G) (there were no obvious changes in the inner retina) (Fig. 1F). These data suggest that the change in total retinal thickness was attributed to the outer retina where the photoreceptors are located

### IBU treatment preserved PR cell function after LD

**Fig. 2:**
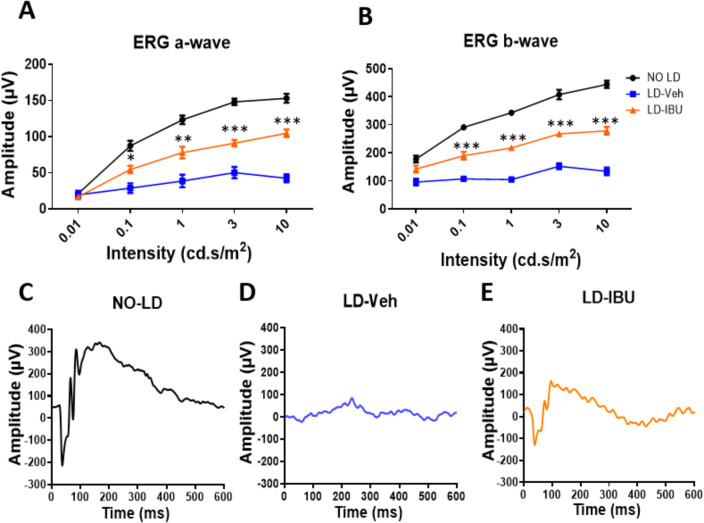
Efficacy of IBU treatment in preserving retinal function following LD. **A-B** Scotopic ERG a-wave and b-wave. Amplitudes of mice ERG with no LD and following LD with either vehicle or IBU 200 mg/kg treatment. *n* = 10 mice per group. **C-E** Representative scotopic ERG recordings of mice with no LD and following LD with either vehicle or IBU treatment

To determine whether systemic IBU treatment could preserve PR function following light-induced retinal damage, we also performed dark-adapted, scotopic ERG analysis on the same groups of mice. IBU-treated mice exhibited significantly higher a-wave and b-wave amplitudes compared to the vehicle-treated group, indicating preservation of retinal function from damaging light exposure (Fig. 2A-E).

### IBU pre-treatment is necessary for retinal protection from damaging light exposure

Our initial IBU treatment paradigm relied on five injections of the drug prior to damaging light exposure, with continued injections for five days post-LD. We wanted to determine how many pre-LD injections were needed and whether pre-LD or post-LD IBU treatment alone could elicit similar retinal protection.

**Fig. 3:**
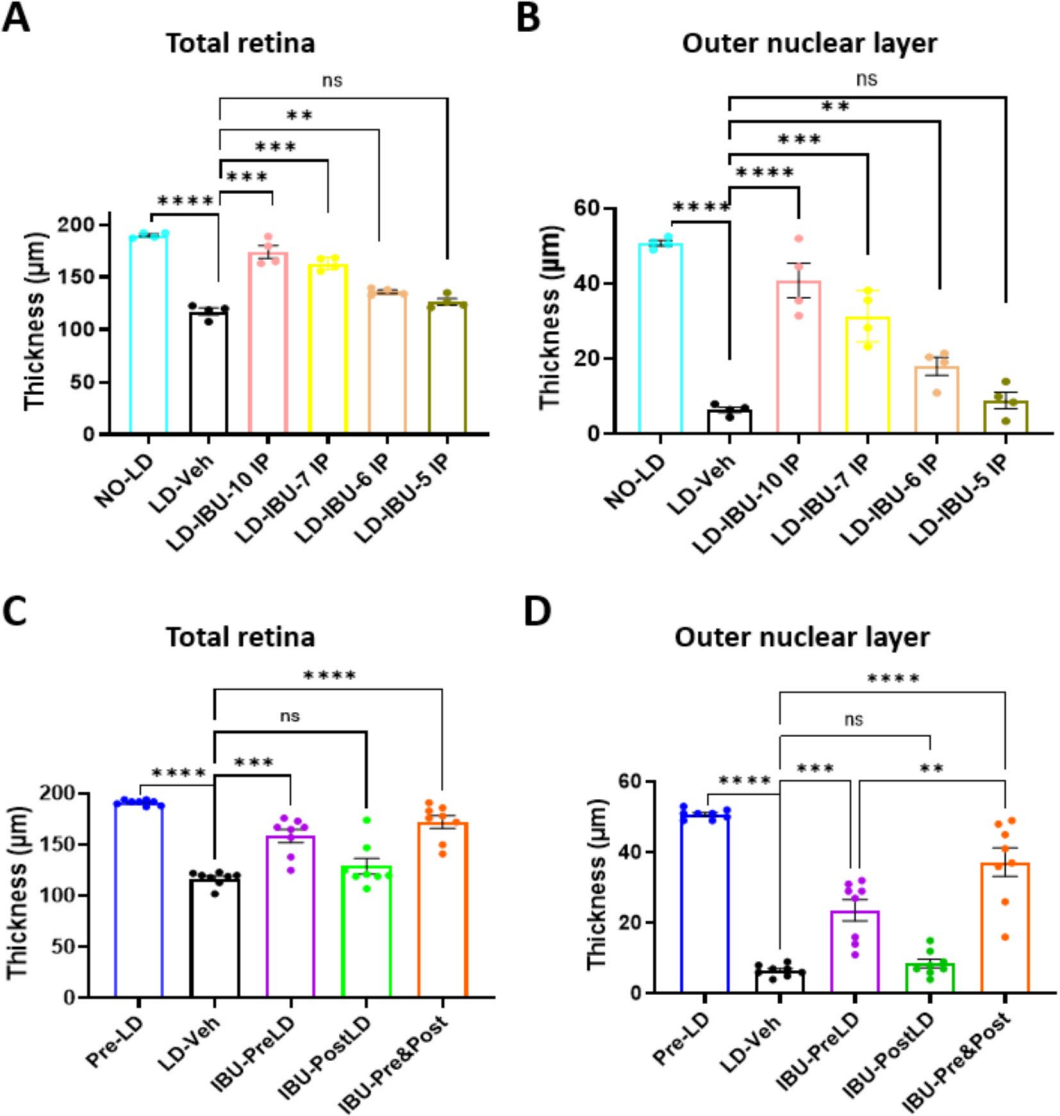
The effects of pre-LD and post-LD IBU treatment on retinal structure measured by SD-OCT. **A-B** Total retina thickness (**A**) and outer nuclear layer thickness (**B**) of mice treated with multiple days of IBU. LD-IBU-10 IP = five IP injections before LD + five IP injections after LD, LD-IBU-7 IP = four IP injections before LD + three IP injections after LD. LD-IBU-6 IP = three IP injections before LD + three IP injections after LD, LD-IBU-5 IP = two IP injections before LD + three IP injections after LD. Dots represent individual mice, *n* = 4 mice per group. **C-D** Differences in the total retinal thickness (**C**) and outer nuclear layer thickness (**D**) with five IBU LD treatments before LD only (IBU-PreLD), five IBU treatments after LD only (IBU-PostLD). Five IP injections were administered before LD and five IP injections after LD (IBU-Pre&Post), *n* = 5 mice per group

Retina swelling was observed from 12 h through day 4 after LD (Figure S1). To test how many days of IBU pre-LD treatment was required for optimal retinal protection, mice were given daily IBU injections for either two, three or four days before damaging light exposure. Results showed that mice required at least four days of IBU treatment before LD for greatest preservation (Fig. 3A-B).

While the combined pre- and post-LD IBU treatment showed the best ONL protection, we found that mice pre-treated with IBU (IBU-PreLD only) showed less ONL thinning than animals receiving only post-LD treatment (IBU-PostLD only) (Fig. 3C-D).

### IBU inhibits upregulation of LD-induced inflammatory factors in retina

Several reports have shown that damaging light exposure induces expression of inflammatory genes [35, 36].

**Fig. 4:**
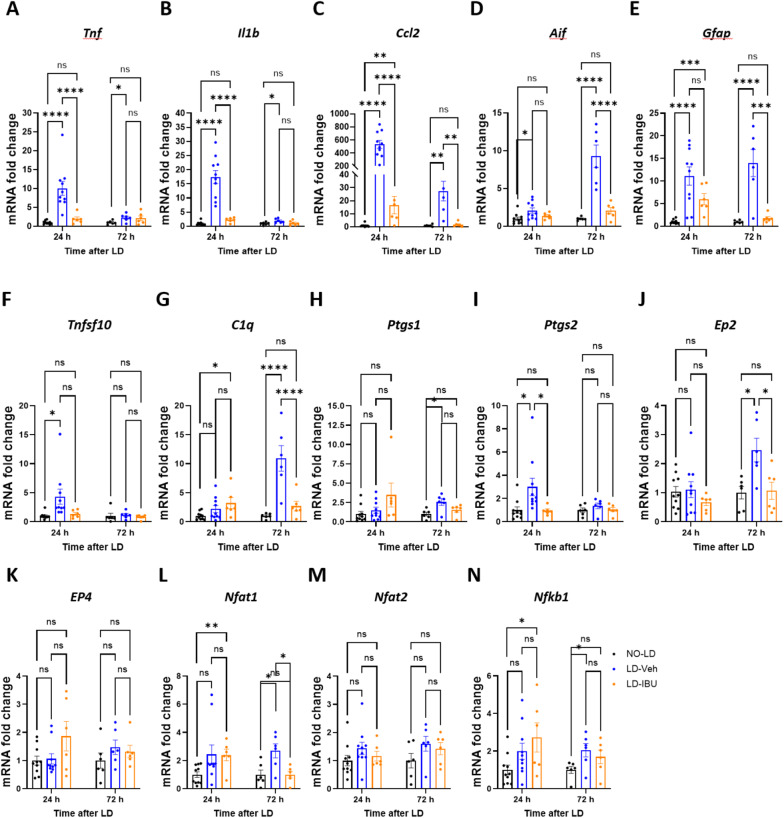
IBU-mediated attenuation of inflammatory factor gene expression in the retina following LD. **A-L** mRNA expression levels of inflammatory factors 24 h and 72 h after LD in mice treated with either vehicle or 200 mg/kg IBU. The qPCR panel measured changes in inflammation-related genes (**A-C**, **F-G**), microglia and Müller cell marker genes (**D-E**), COX and other downstream pathway genes (**H-K**). mRNA expression of immune response transcription factors (**L-N**). Each dot represents individual mouse retina. 5 IP injections were given before LD for all groups (last IP is 2 h before LD). 12 h and 24 h groups had no post LD IP, 72 h groups had 2 IP after LD (same IBU treatment regimen for Figs. 5 and 6). In the 24 h groups, *n* = 10 for NO-LD and LD-Veh and *n* = 6 for LD-IBU. In 72 h groups: *n* = 6

We first check the mRNA expression of inflammatory factors. Our qPCR analysis of mouse retinas revealed that mRNA expression of several inflammatory factors including *Tnf*, *Il1b*, and *Ccl2*, were increased by more than 10, 17 and 530-fold respectively 24 h after LD (Fig. 4A-C), and showed more moderate increases at 72 h post-LD. In 200 mg/kg IBU-treated mice exposed to LD, mRNA expression levels of these three inflammatory factors were reduced to or near the levels of no LD group 24 and 72 h after LD (Fig. 4A-C). Both *Tnfsf10* and *C1q*, genes within the C1q and TNF superfamily involved in inflammation and apoptosis, showed moderate increase 24 h after LD that was inhibited by IBU to the No LD level. At 72 h after LD, the *Tnfsf10* mRNA had no increase. The dramatic increase of *C1q* mRNA at 72 h following LD in vehicle injected mice was reduced to the no LD level in 200 mg/kg IBU injected mice (Fig. 4F-G).

Microglial cells are among the first and the most dominant cell types to respond to neuronal injury. Müller cells interact with microglia and can contribute to inflammatory responses. At 24 h post-LD time point, the mRNA of the general microglia marker *Aif* (Iba1) in the retinas of vehicles showed moderate significant increase while *Gfap* mRNA, which is Müller cell marker gene, showed more dramatic increase. Though both *Aif* and *Gfap* mRNA expressions increased more than those of 24 h in vehicle-treated LD mice and remained at no LD levels in the IBU-treated group (Fig. 4D-E).

IBU’s molecular targets are the cyclooxygenase (COX, formal gene name is *Ptgs*) enzymes [23], specifically *COX-1 (Ptgs1) and COX-2 (Ptgs2)*. IBU is known to inhibit COX-2 and reduce production of prostaglandin E2 (PGE2). At 24 h post LD *Ptgs2* mRNA was significantly upregulated in vehicle-treated mice and remained at no LD levels in IBU-treated mice, while *Ptgs1* expression was unchanged. At 72 h post LD, *Ptgs1* mRNA began to increase in vehicle-treated mice and reduced to no LD level in 200 mg/kg IBU treated mice while *Ptgs2* mRNA expression had no change (Fig. 4H-I). Interestingly, COX-PGE2 pathway related genes, including prostaglandin E receptor2 (*Ep2*) and prostaglandin E receptor 2 (*Ep4*), did not show significant differences in mRNA levels 24 and 72 h after light exposure, with the exception of a significant increase of *Ep2* in vehicle-treated mice which was reduced to no LD level in IBU injected mice (Fig. 4J-K).

MRNA level of the immune response transcription factor *Nfat2* did not show significant change in vehicle-treated LD mice at 24 and 72 h. Upregulations of both *Nfat1* and *Nfkb1* mRNAs 72 h after LD in vehicle-treated mice were reduced to no LD level in IBU-treated mice. (Fig. 4L-N).

### LD induces activation of the necroptosis pathway

**Fig. 5:**
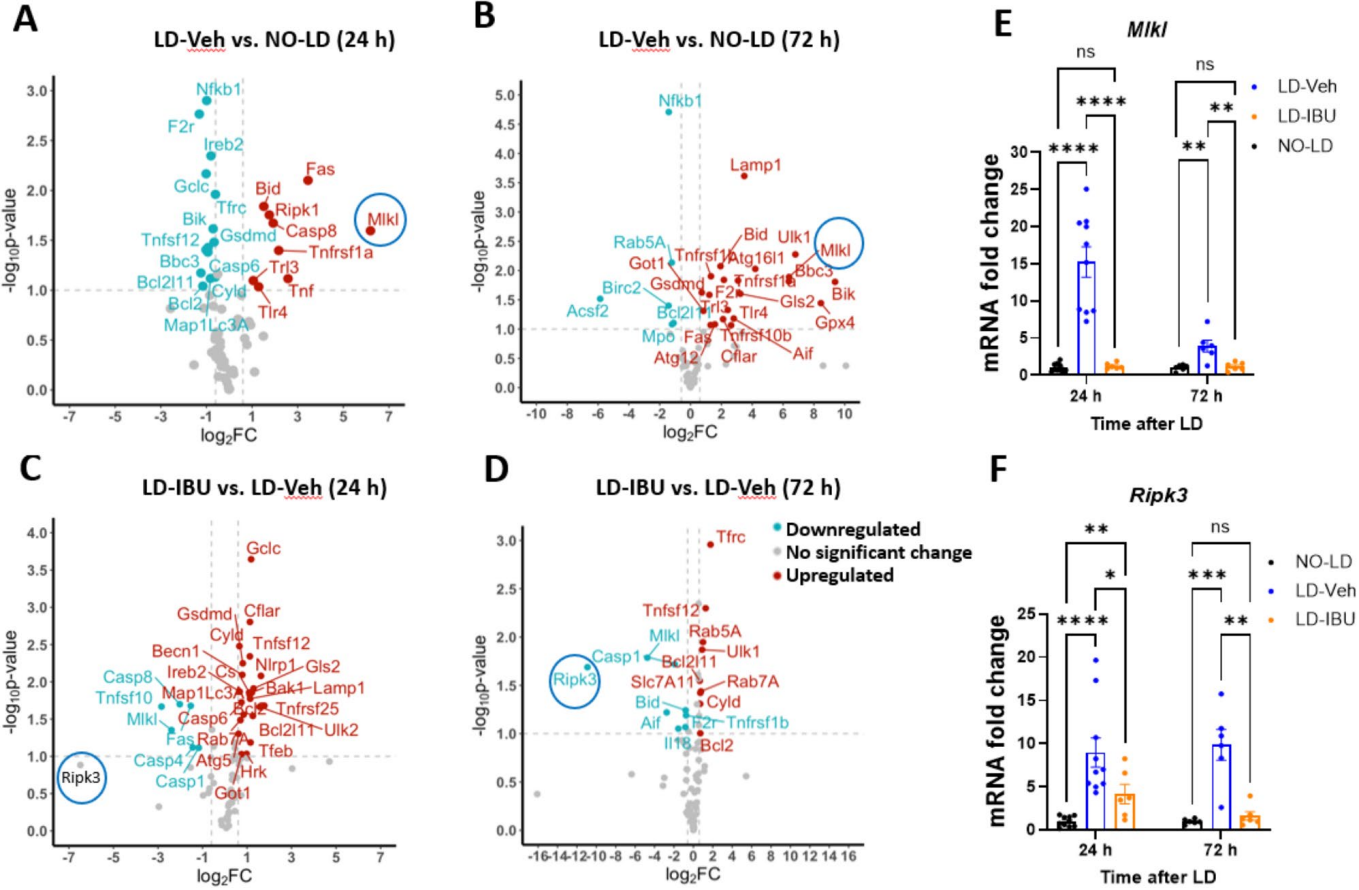
Analysis of LD-induced cell death and IBU inhibition pathways. **A-D** Volcano plots comparing differentially expressed cell death genes in 24 h post-LD vs. no LD mice (**A**), 72 h post-LD vs. no LD mice (**B**), 24 h post-LD mice treated with vehicle vs. 200 mg/kg IBU (**C**), and 72 h post-LD mice treated with vehicle vs. 200 mg/kg IBU (**D**). The x-axis shows log_2_fold change (FC), and the y-axis shows the -log_10_p-value. Upregulated genes (log_2_FC > 0.6 and p-value < 0.1) are shown as red dots, and downregulated genes (log_2_FC < -0.6 and p-value < 0.1) are shown as blue dots. Grey dots indicate genes that were not differentially expressed. **E ***Ripk3* mRNA expression 24 h and 72 h post-LD. **F ***Mlkl* mRNA expression 24 h and 72 h post-LD. Dots represent individual mice. *n* = 8 mice per 24 h groups and *n* = 6 mice per 72 h groups

To explore the mechanism of degeneration associated with light-induced PR loss, we measured the expression of 88 genes involved in different cell death pathways (Table S2) in the mouse retinas of mice not exposed to LD and both 24 h and 72 h post-LD (Fig. 5A). Several genes involved in necroptosis (e.g., *Mlkl*,* Ripk1*,* Trl4*, and *Trl3*) and apoptosis (e.g., *Fas*,* Tnf*,* Tnfrsf1*α, *Casp8*,* Bid*) were upregulated in 24 h post-LD retinas, with *Mlkl* being the most upregulated, showing a 72-fold increase compared to no LD controls (Fig. 5A). In addition, *Ripk3*, an important activator of *Mlkl* in necroptosis, was undetectable in the no LD retinas but was expressed in the LD group, and could therefore be considered upregulated as well (Table S3).

By 72 h post-LD, necroptosis and apoptosis genes still constituted the majority of upregulated genes (Table S4). However, at this time point we also saw upregulation of other cell death pathway genes, including those involved in ferroptosis (e.g., *Gpx4*,* Gls2*,* Got1*), autophagy (e.g., *Ulk1*,* Atg16I1*,* Atg12*), pyroptosis (e.g., *Gsdmd*), methuosis (e.g., *Lamp1*) and parthanatos (e.g., *Aif*,* F2r*) (Fig. 5B), suggesting that additional cell death pathways may also contribute to light-induced PR death.

The Ripk3-Mlkl pathway has been reported to mediate cell membrane rupture-induced necroptosis [37–39]. Upon phosphorylation by *Ripk3*,* Mlkl* undergoes conformational changes and binds to inositol hexaphosphate (*IP6*), which promotes *Mlkl’s* oligomerization and translocation to the plasma membrane, resulting in increased cell membrane permeability and eventual cell death [40–42]. The Ripk3-Mlkl pathway can be triggered by multiple signaling pathways, such as death receptor-Ripk3 pathway activation [43, 44] and toll-like receptor activation (e.g., *Trl3* and *Trl4*) [44, 45]. Here, we found several cell death receptor genes, including *Fas*,* Tnfrsf1a*,* Tnfrsf1b*, and *Tnfrsf10b*, as well as toll-like receptors *Trl3* and *Trl4* were all upregulated in LD retinas, implying that they might promote *Ripk1*-mediated Ripk3-Mlkl and toll-like receptor pathway activation, respectively. Together, these results suggest that necroptosis may play an essential role in LD-induced PR death.

### IBU alleviates upregulation of necrotic cell death genes induced by LD

Based upon our observation of the upregulation of necroptosis-related cell death pathway genes upon induction of LD, we hypothesized that IBU may act to promote PR survival, at least in part, by blocking induction of necroptosis. To examine this hypothesis, we examined the expression of cell death pathway genes in mice treated with or without IBU 24 h and 72 h post-LD. We did not measure the LD-associated increased Mlkl gene expression in the presence of IBU after both 24 and 72 h post injury. We saw a similar effect for the gene encoding cell death receptor, Fas (Fig. 5C, Table S5). In addition, although not statistically significant due to low expression levels in the IBU samples resulting in large variation between samples, Ripk3 was the most downregulated cell death pathway gene in the IBU-treated group. By 72 h, both Ripk3 and Mlkl were the two most significantly down regulated genes in retinas from IBU-treated mice (Fig. 5D, Table S6). We separately confirmed these results by qPCR using independent primer pairs (Fig. 5E-F). These findings suggest that IBU may work, at least partially, through inhibition of the necroptosis pathway to protect PRs from light-induced cell death.

### Upregulated MLKL protein expression and microglia activation in the PR layer of LD retinas

**Fig. 6:**
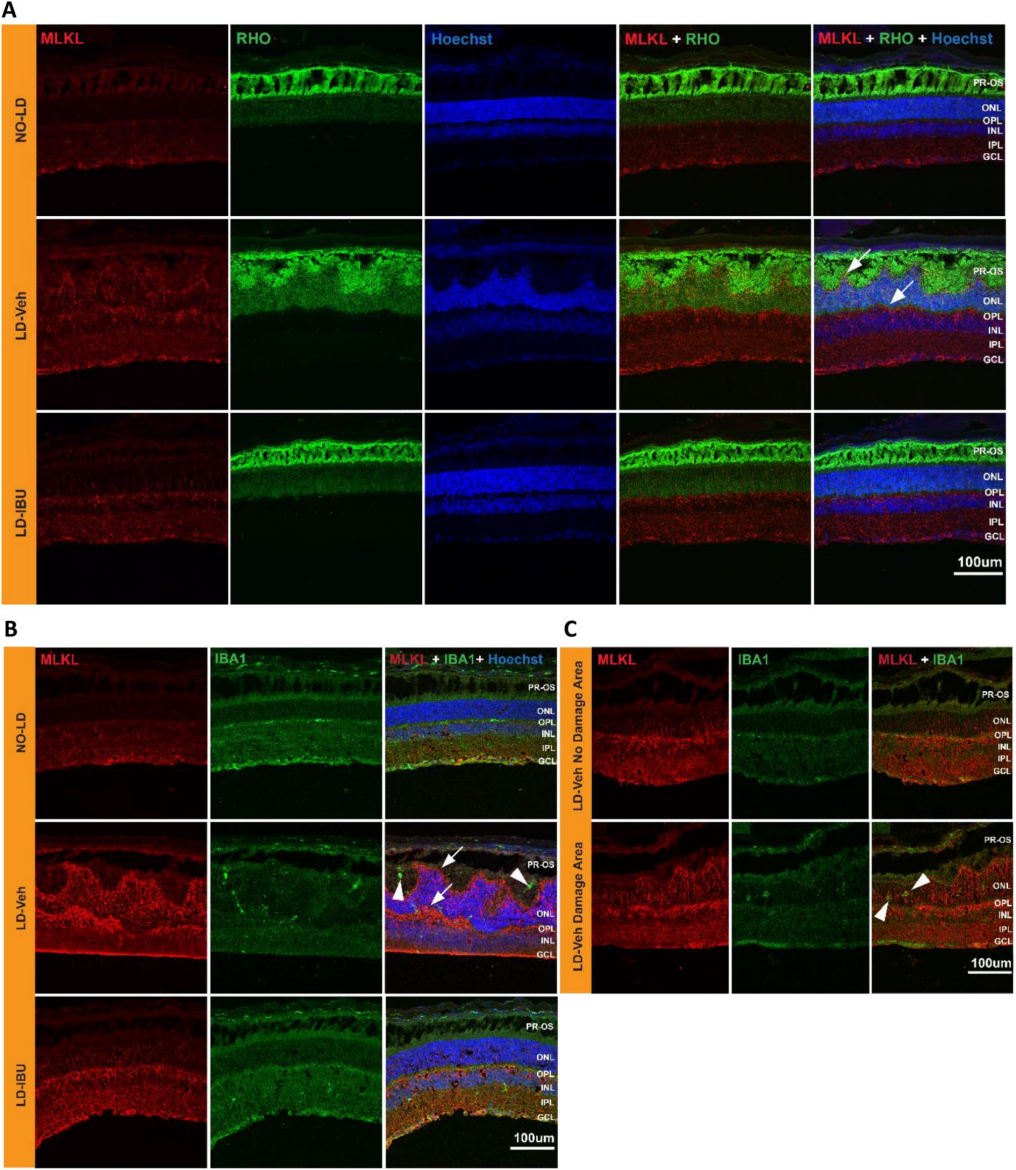
Upregulation of MLKL in retinal PRs and microglia activation at 12 h following LD. **A** IFA images showing MLKL (red) and rhodopsin (RHO, green) protein expression in mouse retinas from NO-LD, LD-Veh and LD-IBU groups. Retinal nuclei were visualized using Hoechst (blue). White arrows indicate MLKL. **B** Anti-MLKL and anti-IBA1 antibodies were co-stained in mouse retinas from NO-LD, LD-Veh and LD-IBU groups. White arrowheads indicate IBA1 protein expression. **C** MLKL and Iba1 expression at the LD area and non-damaged area of a LD-Veh retina. White arrowheads indicate IBA1. Abbreviations: PR-OS, photoreceptor outer segment; ONL, outer nuclear layer; OPL, outer plexiform layer; INL, inner nuclear layer; IPL, inner plexiform layer; GCL, ganglion cell layer

To determine whether the upregulation of necroptosis-related genes measured in LD retina resulted in an increase in proteins they encode, we used immunofluorescence (IFA) to examine retinal sections of no-LD treated, LD-vehicle treated and LD + IBU-treated retinas. IFA revealed MLKL protein expression was increased at 12 h in vehicle-treated LD mice compared to no LD controls. Furthermore, IBU treatment attenuated upregulation of MLKL (Fig. 6A). Upregulation of MLKL was localized to the ONL and OPL layers, the layers that contain PR nuclei and synapses, respectively, indicating that expression was occurring primarily in PR cells (Fig. 6A-B, white arrows), supporting the hypothesis that necroptosis plays a role in LD-induced PR injury and cell death [46].

Microglia activation and migration were also observed in the LD retinas, which was alleviated in the IBU-treated mice. We found microglia, which migrate to the site of injury to clear damaged cells, in the ONL of LD retinas (Fig. 6B, white arrowheads). There was no microglia migration to the ONL in mice not exposed to LD nor in IBU-protected mouse retinas (Fig. 6B). Microglia activation and migration was observed in the damaged areas of the retina and not in non-damaged areas of the same LD retina, even in the same vehicle-treated LD mice (Fig. 6C, white arrowheads). As assessed by staining with anti-GFAP and anti-MLKL antibodies, we did not observe evidence of Müller cell activation (Figure S3).

## Discussion

We found that systemic administration of the anti-inflammatory drug IBU protects mouse retinas from light-induced photochemical damage. We also found that MLKL-mediated necroptosis plays a role in PR death following LD, and that IBU inhibits the light-mediated increase in MLKL expression. In this study we used cool-fluorescent light to induce photochemical damage to albino mouse retinas. A possible mechanism of photochemical damage to PRs involves overproduction of retinoid cycle by-products induced by high-intensity light exposure inducing PR and retinal pigment epithelial (RPE) cell stress and eventual cell death [47]. RPE death was not obvious in our LD experiments at 72 h (Figure S4), and the PR degeneration in our LD experiments was quicker (as early as 12 h) and more severe than observed in some other published studies [48, 49]. In our model, it seems that PR cells, rather than RPE, are the major direct target of light-induced retinal stress.

The outer plexiform layer (OPL) consists of the neuronal synaptic connections among photoreceptors, bipolar and horizontal cells. Müller cells provide structural support and maintain the extracellular environment for these cells. The outer nuclear layer (ONL) of the retina contains the nuclei of photoreceptor cells. MLKL upregulation in the ONL after LD was reduced by IBU and, interestingly, upregulation in the OPL was not affected by IBU treatment. This suggests that IBU’s inhibition effect may be cell type and/or pathway specific.

It’s known that activated microglia and Müller cells secrete inflammatory molecules that can accelerate and worsen PR degeneration [50], and it has been shown that anti-inflammatory treatments can reduce PR loss in several retinal degeneration models [13, 14, 51]. Here, we provided direct evidence that systemic administration of IBU can protect mouse PRs, maintaining 75% of ONL thickness and preserving ERG function, two weeks after damaging light exposure. IBU inhibited the upregulation of *Tnf* and *Il1b* mRNA expression that occurs after LD, leading to levels comparable to mice not exposed to LD. We found that expression of *Ccl2*, an inflammatory gene predominantly expressed by in the retina Müller cells [52, 53], showed more than 530-fold increased expression 24 h after LD, while IBU treatment completely prevented this upregulation.

Our results showed that retinal structure was better preserved with IBU pre-LD treatment compared with post-LD treatment. This suggests that IBU treatment has a preventive effect for acute LD-induced photoreceptor damage. Though dry AMD and retinitis pigmentosa usually progress slowly over years, with chronic and progressive cell injury, and light damage is an acute photoreceptor injury, these findings suggest that if IBU turns out to be useful for treatment of dry AMD and/or retinitis pigmentosa, it may be desirable to initiate treatment as soon as possible.

Our results are also consistent with previous reports that show that PR protection can be achieved by steroids and other drugs through targeting anti-inflammation pathways and inhibiting cytokines such as TNF and IL1B, which play a key role in inducing inflammation. Further supporting our data, Kim et al. showed that an hyaluronic acid-based inflammation-responsive hydrogel reduced inflammation-related gene expression and attenuated outer retinal degeneration in the rd10 RP mouse model [51]. IBU has the advantage of being an FDA-approved and widely used medication that has few side effects, which is ideal for repurposed clinical use.

Several studies have suggested that PR apoptosis is the major cell death pathway contributing to light-induced degeneration [46]. We discovered that the gene expression of members of the Ripk3-Mlkl pathway, a mediator of cell membrane rupture-induced necroptosis, were dramatically changed after at LD, both at 24 and 72 h after LD, and. Our data showing that IBU suppresses those changes suggests that the IBU-mediated PR protection may be mediated, at least in part, through inhibition of the Ripk3-Mlkl necroptosis pathway in stressed PR cells exposed to LD. In our study, IBU treatment also suppressed the effect on LD induced increased expression of both inflammation- and necroptosis-related genes. Whether or not IBU’s neuroprotective activity is due to a direct effect on PRs (perhaps through direct inhibition of the Ripk3-Mlkl necroptosis), or due to an indirect effect on non-PR cells (perhaps through inhibiting the release of inflammatory factors from retinal glial) is unclear (Figure S5). Based on the report that microglia depletion/repopulation does not affect light-induced retinal degeneration in mice [54], it seems that microglia’s contribution to light exposure-caused inflammation is likely less than other immune cells. Further studies will be needed to answer this and other questions about the mechanisms by which IBU, and presumably other immune modulators, promotes PR survival and function.

## Conclusion

Systemic administration of the anti-inflammatory drug IBU protected mouse photoreceptors from light-induced damage. Further analysis showed that IBU reduced expression of inflammatory factors and attenuated necrotic photoreceptor cell death by *Ripk3* and *Mlkl*. These findings provide new information relevant to the treatment of the human retinal degenerative diseases.

## Electronic supplementary material

Below is the link to the electronic supplementary material.

Supplementary Material 1

## Data Availability

No datasets were generated or analysed during the current study.

## Abbreviations

Aif: Allograft inflammatory factor 1
AMD: Age-related macular degeneration
ARVO: Association for Research in Vision and Ophthalmology
Atg12: Autophagy Related 12
Atg16I1: Autophagy Related 16 Like 1
Bid: BH3 Interacting Domain Death Agonist
C1q: Complement C1q Chain
Casp8: Cysteine-aspartic acid protease (caspase) family 8
Ccl2: Chemokine (C-C motif) ligand 2
Cox-1: Cyclooxygenase-1
Cox-2: Cyclooxygenase-2
DMSO: Dimethyl sulfoxide
ELM: External limiting membrane
Ep2: PTGER2
Ep4: PTGER4
ERG: Electroretinography
F2r: Coagulation Factor II Thrombin Receptor
FDA: Food and Drug Administration
GCL: Ganglion cell layer
Gfap: Glial fibrillary acidic protein
Gls2: Glutaminase 2
Got1: Glutamic-Oxaloacetic Transaminase 1
Gpx4: Glutathione peroxidase 4
Gsdmd: Gasdermin D
H&E staining: Hematoxylin and eosin staining
IACUC: Institutional Animal Care and Use Committee
IBU: Ibuprofen
IC: Inferior central
IE: Inferior equatorial
Il1B: Interleukin 1 beta
ILM: Internal limiting membrane
INL: Inner nuclear layer
IP: Inferior peripheral
IP: Intraperitoneal injection
IPL: Inner plexiform layer
IS: Inner segment of the photoreceptors
Lamp1: Lysosomal-associated membrane protein 1
LD: Light damage
Mlkl: Mixed Lineage Kinase Domain Like Pseudokinase
Nfat-1: Nuclear factor of activated T-cells 1
Nfat-2: Nuclear factor of activated T-cells 2
NSAID: Nonsteroidal anti-inflammatory drug
ONL: Outer nuclear layer
OPL: Outer plexiform layer
OS: Outer segment of the photoreceptors
PR: Photoreceptor
Ptges: Prostaglandin E synthase
RT: Reverse Transcription
qPCR: Quantitative PCR
Ripk3: Receptor Interacting Serine/Threonine Kinase 3
RNAse: Ribonuclease
RNFL: Retinal nuclear fiber layer
RP: Retinitis pigmentosa
RPE: Retinal pigment epithelial
SC: Superior central
SD-OCT: Spectral-domain optical coherence tomography
SE: Superior equatorial
SP: Superior peripheral
Tnf: Tumor Necrosis Factor
Tnfsf10: Tumor Necrosis Factor (ligand) Superfamily, Member 10
Tnf: Tumor necrosis factor-alpha
Trl3: Toll-like receptor 3
Trl4: Toll-like receptor 4
Ulk1: Unc-51 Like Autophagy Activating Kinase 1
