# Supplementary material for: Ibuprofen reduces inflammation, necroptosis and protects photoreceptors from light-induced retinal degeneration
Source: J Neuroinflammation. 2025 Jan 28;22:20. doi: 10.1186/s12974-024-03329-8 (PMC11773948; doi:10.1186/s12974-024-03329-8)
Supplement: Supplementary file 1 — Supplementary Material 1 [file 12974_2024_3329_MOESM1_ESM.docx]

**Table S1. qPCR primers**

| mTnf-F | GGTGCCTATGTCTCAGCCTCTT |
| --- | --- |
| mTnf-R | GCCATAGAACTGATGAGAGGGAG |
| mIl1b-F | GAAATGCCACCTTTTGACAGTG |
| mIl1b-R | TGGATGCTCTCATCAGGACAG |
| mCcl2-F | CATCCACGTGTTGGCTCA |
| mCcl2-R | TCTGGACCCATTCCTTCTTG |
| mAif1-F | TCTGCCGTCCAAACTTGAAGCC |
| mAif1-R | CTCTTCAGCTCTAGGTGGGTCT |
| mGfap-F | CCCTGGCTCGTGTGGATTT |
| mGfap-R | GACCGATACCACTCCTCTGTC |
| mTnfsf10q-F | GGAAGACCTCAGAAAGTGGCAG |
| mTnfsf10q-R | TTTCCGAGAGGACTCCCAGGAT |
| mC1q-F | GTGGCTGAAGATGTCTGCCGAG |
| mC1q-R | TTAAAACCTCGGATACCAGTCCG |
| mCox1-qF | GAATGCCACCTTCATCCGAGAAG |
| mCox1-qR | GCTCACATTGGAGAAGGACTCC |
| mCox2-F | GCGACATACTCAAGCAGGAGCA |
| mCox2-R | AGTGGTAACCGCTCAGGTGTTG |
| mPtges-F | CTGGTGATGGAGAGCGGCC |
| mPtges-R | GCGATCAGAGGGTTGGGTCC |
| mEp2-F | CTGCGGATTGTCTGGCAGTAG |
| mEp2-R | GAGCTCGGAGGTCCCACTTTT |
| mEp4-F | ACTGCGTGGGAAGAGACTGA |
| mEp4-R | GGATGGGGTTCACAGAAGCA |
| mNfat1-F | GGTGCCTTTTGCGAGCAGTATC |
| mNfat1-R | CGTATGGACCAGAATGTGACGG |
| mNfat2-F | ACTTCACAGCGGAGTCCAAGGT |
| mNfat2-R | GGATGTGCTTGTTCCGATACTCG |
| mNfkb1-F | GCTGCCAAAGAAGGACACGACA |
| mNfkb1-R | GGCAGGCTATTGCTCATCACAG |
| mRipk3-qF | GAAGACACGGCACTCCTTGGTA |
| mRipk3-qR | CTTGAGGCAGTAGTTCTTGGTGG |
| mMlkl-F | CTGAGGGAACTGCTGGATAGAG |
| mMlkl-R | CGAGGAAACTGGAGCTGCTGAT |
| mGapdh-F | TCAACAGCAACTCCCACTCTTCCA |
| mGapdh-R | ACCCTGTTGCTGTAGCCGTATTCA |

**Table S2. Pan-Cell Death Pathway PCR Array gene list**

| **Gene symbol** | **Gene Name** | **Annotation** |
| --- | --- | --- |
| *Bad* | BCL2-associated agonist of cell death | Apoptosis-Intrinsic |
| *Bak1* | BCL2-antagonist/killer 1 | Apoptosis-Intrinsic |
| *Bax* | BCL2-associated X protein | Apoptosis-Intrinsic |
| *Bbc3* | BCL2 binding component 3 | Apoptosis-Intrinsic |
| *Bcl2* | B-cell CLL/lymphoma 2 | Apoptosis-Intrinsic |
| *Bcl2l11* | BCL2-like 1 | Apoptosis-Intrinsic |
| *Bid* | BH3 interacting domain death agonist | Apoptosis-Intrinsic |
| *Bik* | BCL2-interacting killer (apoptosis-inducing) | Apoptosis-Intrinsic |
| *Bcl2l11* | BCL2-like 11 | Apoptosis-Intrinsic |
| *Casp9* | caspase 9, apoptosis-related cysteine peptidase | Apoptosis-Intrinsic |
| *Hrk* | harakiri, BCL2 interacting protein | Apoptosis-Intrinsic |
| *Mcl1* | myeloid cell leukemia 1 | Apoptosis-Intrinsic |
| *Casp8* | caspase 8, apoptosis-related cysteine peptidase | Apoptosis-Extrinsic |
| *Fas* | Fas cell surface death receptor | Apoptosis-Extrinsic |
| *Faslg* | Fas ligand (TNF superfamily, member 6) | Apoptosis-Extrinsic |
| *Tnf* | tumor necrosis factor | Apoptosis-Extrinsic |
| *Tnfrsf10a* | tumor necrosis factor receptor superfamily, member 10a | Apoptosis-Extrinsic |
| *Tnfrsf10b* | tumor necrosis factor receptor superfamily, member 10b | Apoptosis-Extrinsic |
| *Tnfrsf10c* | tumor necrosis factor receptor superfamily, member 10c, decoy without an intracellular domain | Apoptosis-Extrinsic |
| *Tnfrsf1a* | tumor necrosis factor receptor superfamily, member 1a | Apoptosis-Extrinsic |
| *Tnfrsf1b* | tumor necrosis factor receptor superfamily, member 1b | Apoptosis-Extrinsic |
| *Tnfrsf25* | tumor necrosis factor receptor superfamily, member 25 | Apoptosis-Extrinsic |
| *Tnfsf10* | tumor necrosis factor (ligand) superfamily, member 10 | Apoptosis-Extrinsic |
| *Tnfsf12* | tumor necrosis factor (ligand) superfamily, member 12 | Apoptosis-Extrinsic |
| *Atg12* | autophagy related 12 | Autophagy |
| *Atg14* | autophagy related 14 | Autophagy |
| *Atg16l1* | autophagy related 16-like 1 (S. cerevisiae) | Autophagy |
| *Atg5* | autophagy related 5 | Autophagy |
| *Atg7* | autophagy related 7 | Autophagy |
| *Becn1* | beclin 1, autophagy related | Autophagy |
| *Gabarap* | GABA(A) receptor-associated protein | Autophagy |
| *Map1lc3a* | microtubule Associated Protein 1 Light Chain 3 alpha | Autophagy |
| *Sqstm1* | sequestosome 1 | Autophagy |
| *Tfeb* | transcription factor EB | Autophagy |
| *Ulk1* | unc-51 like autophagy activating kinase 1 | Autophagy |
| *Ulk2* | unc-51 like autophagy activating kinase 2 | Autophagy |
| *Acsf2* | acyl-CoA synthetase family member 2 | Ferroptosis |
| *Atp5g3* | ATP synthase, H+ transporting, mitochondrial complex V, subunit C3 (subunit 9) | Ferroptosis |
| *Cs* | citrate synthase | Ferroptosis |
| *Gclc* | glutamate-cysteine ligase, catalytic subunit | Ferroptosis |
| *Gls2* | glutaminase 2 (liver, mitochondrial) | Ferroptosis |
| *Got1* | glutamic-oxaloacetic transaminase 1, soluble | Ferroptosis |
| *Gpx4* | glutathione peroxidase 4 | Ferroptosis |
| *Ireb2* | iron-responsive element binding protein 2 | Ferroptosis |
| *Rpl8* | ribosomal protein L8 | Ferroptosis |
| *Slc1a5* | solute carrier family 1 (neutral amino acid transporter), member 5 | Ferroptosis |
| *Slc7a11* | solute carrier family 7 (anionic amino acid transporter light chain, xc- system), member 11 | Ferroptosis |
| *Tfrc* | transferrin receptor | Ferroptosis |
| *Birc2* | baculoviral IAP repeat containing 2 | Necroptosis |
| *Birc3* | baculoviral IAP repeat containing 3 | Necroptosis |
| *Cflar* | CASP8 and FADD-like apoptosis regulator | Necroptosis |
| *Cyld* | cylindromatosis (turban tumor syndrome) | Necroptosis |
| *Fadd* | Fas (TNFRSF6)-associated via death domain | Necroptosis |
| *Mlkl* | mixed lineage kinase domain-like | Necroptosis |
| *Ripk1* | receptor (TNFRSF)-interacting serine-threonine kinase 1 | Necroptosis |
| *Ripk3* | receptor-interacting serine-threonine kinase 3 | Necroptosis |
| *Tlr3* | toll-like receptor 3 | Necroptosis |
| *Tlr4* | toll-like receptor 4 | Necroptosis |
| *Tradd* | TNFRSF1A-associated via death domain | Necroptosis |
| *Traf2* | TNF receptor-associated factor 2 | Necroptosis |
| *Aim2* | absent in melanoma 2 | Pyroptosis |
| *Casp1* | caspase 1, apoptosis-related cysteine peptidase | Pyroptosis |
| *Lamp2* | lysosomal-associated membrane protein 2 | Pyroptosis |
| *Casp4* | caspase 4, apoptosis-related cysteine peptidase | Pyroptosis |
| *Casp5* | caspase 5, apoptosis-related cysteine peptidase | Pyroptosis |
| *Gsdmd* | gasdermin D | Pyroptosis |
| *Il18* | interleukin 18 | Pyroptosis |
| *Il1b* | interleukin 1, beta | Pyroptosis |
| *Nfkb1* | nuclear factor of kappa light polypeptide gene enhancer in B-cells 1 | Pyroptosis |
| *Nlrc4* | NLR family, CARD domain containing 4 | Pyroptosis |
| *Nlrp1* | NLR family, pyrin domain containing 1 | Pyroptosis |
| *Nlrp3* | NLR family, pyrin domain containing 3 | Pyroptosis |
| *Elane* | elastase, neutrophil expressed | NETosis |
| *Ctsg* | cathepsin G | NETosis |
| *Azu1* | azurocidin 1 | NETosis |
| *Mpo* | myeloperoxidase | NETosis |
| *Padi4* | peptidyl Arginine Deiminase 4 | NETosis |
| *Cdh1* | cadherin 1, type 1, E-cadherin (epithelial) | Entosis |
| *Cdh3* | cadherin 3, type 1, P-cadherin (placental) | Entosis |
| *Rhoa* | ras homolog family member A | Entosis |
| *Hras* | Harvey rat sarcoma viral oncogene homolog | Methuosis |
| *Lamp1* | lysosomal-associated membrane protein 1 | Methuosis |
| *Rab5a* | RAB5A, Member RAS Oncogene Family | Methuosis |
| *Rab7a* | RAB7A, Member RAS Oncogene Family | Methuosis |
| *Aif* | allograft inflammatory factor 1 | Parthanatos |
| *Mif* | macrophage Migration Inhibitory Factor | Parthanatos |
| *Par* | PAR protein | Parthanatos |
| *Parg* | poly (ADP-ribose) glycohydrolase | Parthanatos |
| *Actb* | Actin, beta | Housekeeping Genes |
| *B2m* | Beta-2-microglobulin | Housekeeping Genes |
| *Gapd* | Glyceraldehyde-3-phosphate dehydrogenase | Housekeeping Genes |
| *Gusb* | Glucuronidase, beta | Housekeeping Genes |
| *Hprt1* | Hypoxanthine phosphoribosyltransferase 1 | Housekeeping Genes |
| *Pgk* | Phosphoglycerate kinase 1 | Housekeeping Genes |
| *Ppia* | Peptidylprolyl isomerase A | Housekeeping Genes |
| *Rpl13a* | Ribosomal protein L13a | Housekeeping Genes |

| **Table S3. Gene expression changes in LD-Vehicle retina vs. no LD at 24 hrs** |  |
| --- | --- |

| **Gene** | **Annotation** | **Fold Change** | **P value** | **Direction** |
| --- | --- | --- | --- | --- |
| *Mlkl* | Necroptosis | 72.99506 | 0.025392 | **UP** |
| *Fas* | Apoptosis-extrinsic | 10.94703 | 0.007938 | **UP** |
| *Tnf* | Apoptosis-extrinsic | 5.963991 | 0.076745 | **UP** |
| *Tnfrsf1a* | Apoptosis-extrinsic | 4.496509 | 0.040075 | **UP** |
| *Casp8* | Apoptosis-extrinsic | 3.800598 | 0.021292 | **UP** |
| *Ripk1* | Necroptosis | 3.376376 | 0.017621 | **UP** |
| *Bid* | Apoptosis-intrinsic | 2.85775 | 0.01449 | **UP** |
| *Tlr4* | Necroptosis | 2.443729 | 0.092626 | **UP** |
| *Trl3* | Necroptosis | 2.075707 | 0.080269 | **UP** |
| *F2r* | Parthanatos | 0.402668 | 0.00172 | **DOWN** |
| *Bcl2l11* | Apoptosis-intrinsic | 0.422629 | 0.06729 | **DOWN** |
| *Bcl2* | Apoptosis-intrinsic | 0.448642 | 0.09112 | **DOWN** |
| *Bbc3* | Apoptosis-intrinsic | 0.493631 | 0.039446 | **DOWN** |
| *Gclc* | Ferroptosis | 0.495606 | 0.006825 | **DOWN** |
| *Nfkb1* | Pyroptosis | 0.503991 | 0.001259 | **DOWN** |
| *Casp6* | Pyroptosis | 0.522926 | 0.041614 | **DOWN** |
| *Tnfsf12* | Apoptosis-extrinsic | 0.526821 | 0.037312 | **DOWN** |
| *Map1lc3a* | Autophagy | 0.564335 | 0.076412 | **DOWN** |
| *Ireb2* | Ferroptosis | 0.573807 | 0.00451 | **DOWN** |
| *Bik* | Apoptosis-intrinsic | 0.612565 | 0.024268 | **DOWN** |
| *Gsdmd* | Pyroptosis | 0.631696 | 0.033057 | **DOWN** |
| *Cyld* | Necroptosis | 0.652641 | 0.074189 | **DOWN** |
| *Tfrc* | Ferroptosis | 0.657633 | 0.01097 | **DOWN** |

**Table S4, Gene expression changes in LD-Vehicle retina vs. No-LD at 72hrs**

| **Gene** | **Annotation** | **Fold Change** | **P value** | **Direction** |
| --- | --- | --- | --- | --- |
| *Bik* | Apoptosis-intrinsic | 662.7828 | 0.015589 | **UP** |
| *Gpx4* | Ferroptosis | 350.2738 | 0.035994 | **UP** |
| *Ulk1* | Autophagy | 111.2123 | 0.005297 | **UP** |
| *Mlkl* | Necroptosis | 83.66036 | 0.012878 | **UP** |
| *Bbc3* | Apoptosis-intrinsic | 82.62739 | 0.015283 | **UP** |
| *Atg16l1* | Autophagy | 18.34101 | 0.009364 | **UP** |
| *Lamp1* | Methuosis | 11.18304 | 0.000242 | **UP** |
| *Gls2* | Ferroptosis | 9.353191 | 0.024919 | **UP** |
| *Tnfrsf1a* | Apoptosis-extrinsic | 8.434012 | 0.014842 | **UP** |
| *Aif* | Parthanatos | 6.961552 | 0.065826 | **UP** |
| *Tnfrsf10b* | Apoptosis-extrinsic | 6.184281 | 0.085988 | **UP** |
| *Tlr4* | Necroptosis | 5.324468 | 0.047053 | **UP** |
| *F2r* | Parthanatos | 4.472848 | 0.014437 | **UP** |
| *Cflar* | Necroptosis | 4.312494 | 0.067588 | **UP** |
| *Bid* | Apoptosis-intrinsic | 3.840675 | 0.008415 | **UP** |
| *Atg12* | Autophagy | 2.97817 | 0.082856 | **UP** |
| *Tnfrsf1b* | Apoptosis-extrinsic | 2.498622 | 0.012508 | **UP** |
| *Fas* | Apoptosis-extrinsic | 2.463814 | 0.084982 | **UP** |
| *Trl3* | Necroptosis | 2.326312 | 0.02598 | **UP** |
| *Got1* | Ferroptosis | 1.783193 | 0.048793 | **UP** |
| *Gsdmd* | Pyroptosis | 1.653095 | 0.02373 | **UP** |
| *Acsf2* | Ferroptosis | 0.017184 | 0.030367 | **DOWN** |
| *Birc2* | Necroptosis | 0.371841 | 0.039864 | **DOWN** |
| *Nfkb1* | Pyroptosis | 0.375327 | 1.9586799226774e-05 | **DOWN** |
| *Rab5a* | Methuosis | 0.429739 | 0.007314 | **DOWN** |
| *Mpo* | NETosis | 0.434096 | 0.082659 | **DOWN** |
| *Bcl2l11* | Apoptosis-intrinsic | 0.465881 | 0.078514 | **DOWN** |

**Table S5, Gene expression changes in LD-IBU retina vs. LD-Vehicle at 24hrs**

| **Gene** | **Annotation** | **Fold change** | **P value** | **Direction** |
| --- | --- | --- | --- | --- |
| *Tnfsf10* | Apoptosis-extrinsic | 0.139431 | 0.021507 | **DOWN** |
| *Mlkl* | Necroptosis | 0.190133 | 0.044345 | **DOWN** |
| *Casp8* | Apoptosis-extrinsic | 0.246377 | 0.019958 | **DOWN** |
| *Fas* | Apoptosis-extrinsic | 0.346558 | 0.020996 | **DOWN** |
| *Casp4* | Pyroptosis | 0.369312 | 0.07594 | **DOWN** |
| *Casp1* | Pyroptosis | 0.444894 | 0.076668 | **DOWN** |
| *Tnfrsf25* | Apoptosis-extrinsic | 3.422282 | 0.020897 | **UP** |
| *Nlrp1* | Pyroptosis | 3.082297 | 0.008321 | **UP** |
| *Ulk2* | Autophage | 2.952654 | 0.021685 | **UP** |
| *Gls2* | Ferroptosis | 2.451357 | 0.012281 | **UP** |
| *Bcl2l11* | Apoptosis-intrinsic | 2.396515 | 0.028701 | **UP** |
| *Bak1* | Apoptosis-intrinsic | 2.323933 | 0.013937 | **UP** |
| *Gclc* | Ferroptosis | 2.271953 | 0.000227 | **UP** |
| *Tfeb* | Autophage | 2.22776 | 0.065168 | **UP** |
| *Lamp1* | Methuosis | 2.201182 | 0.016858 | **UP** |
| *Cflar* | Necroptosis | 2.187675 | 0.001573 | **UP** |
| *Tnfsf12* | Apoptosis-extrinsic | 2.181222 | 0.004552 | **UP** |
| *Map1Lc3a* | Autophage | 2.171287 | 0.014959 | **UP** |
| *Cs* | Ferroptosis | 2.13496 | 0.013932 | **UP** |
| *Hrk* | Apoptosis-intrinsic | 1.974409 | 0.092787 | **UP** |
| *Bcl2* | Apoptosis-intrinsic | 1.7967 | 0.027539 | **UP** |
| *Gsdmd* | Pyroptosis | 1.750667 | 0.005645 | **UP** |
| *Becn1* | Autophage | 1.723865 | 0.008056 | **UP** |
| *Got1* | Ferroptosis | 1.681668 | 0.093527 | **UP** |
| *Casp6* | Pyroptosis | 1.678937 | 0.018833 | **UP** |
| *Rab7a* | Methuosis | 1.626856 | 0.032685 | **UP** |
| *Ireb2* | Ferroptosis | 1.587965 | 0.013534 | **UP** |
| *Cyld* | Necroptosis | 1.575352 | 0.003317 | **UP** |
| *Atg5* | Autophage | 1.534139 | 0.049835 | **UP** |

**Table S6. Gene expression changes in LD-IBU retina vs. LD-Vehicle at 72 hrs**

| **Gene** | **Annotation** | **Fold change** | **P value** | **Direction** |
| --- | --- | --- | --- | --- |
| *Ripk3* | Necroptosis | 0.000518 | 0.020492 | **DOWN** |
| *Mlkl* | Necroptosis | 0.037359 | 0.016346 | **DOWN** |
| *Aif* | Parthanatos | 0.148447 | 0.060279 | **DOWN** |
| *Casp1* | Pyroptosis | 0.262307 | 0.019093 | **DOWN** |
| *Il18* | Pyroptosis | 0.34183 | 0.088726 | **DOWN** |
| *F2r* | Parthanatos | 0.565956 | 0.085477 | **DOWN** |
| *Bid* | Apoptosis-intrinsic | 0.575337 | 0.057184 | **DOWN** |
| *Tnfrsf1b* | Apoptosis-extrinsic | 0.592672 | 0.064641 | **DOWN** |
| *Bcl2* | Apoptosis-intrinsic | 1.637877 | 0.099212 | **UP** |
| *Bcl2l11* | Apoptosis-intrinsic | 1.572651 | 0.028354 | **UP** |
| *Tnfsf12* | Apoptosis-extrinsic | 2.403763 | 0.005034 | **UP** |
| *Ulk1* | Autophage | 1.869067 | 0.013513 | **UP** |
| *Slc7a11* | Ferroptosis | 1.644828 | 0.037839 | **UP** |
| *Tfrc* | Ferroptosis | 3.395455 | 0.001104 | **UP** |
| *Cyld* | Necroptosis | 1.622023 | 0.049147 | **UP** |
| *Rab5a* | Methuosis | 1.988773 | 0.011289 | **UP** |
| *Rab7a* | Methuosis | 1.720208 | 0.036464 | **UP** |


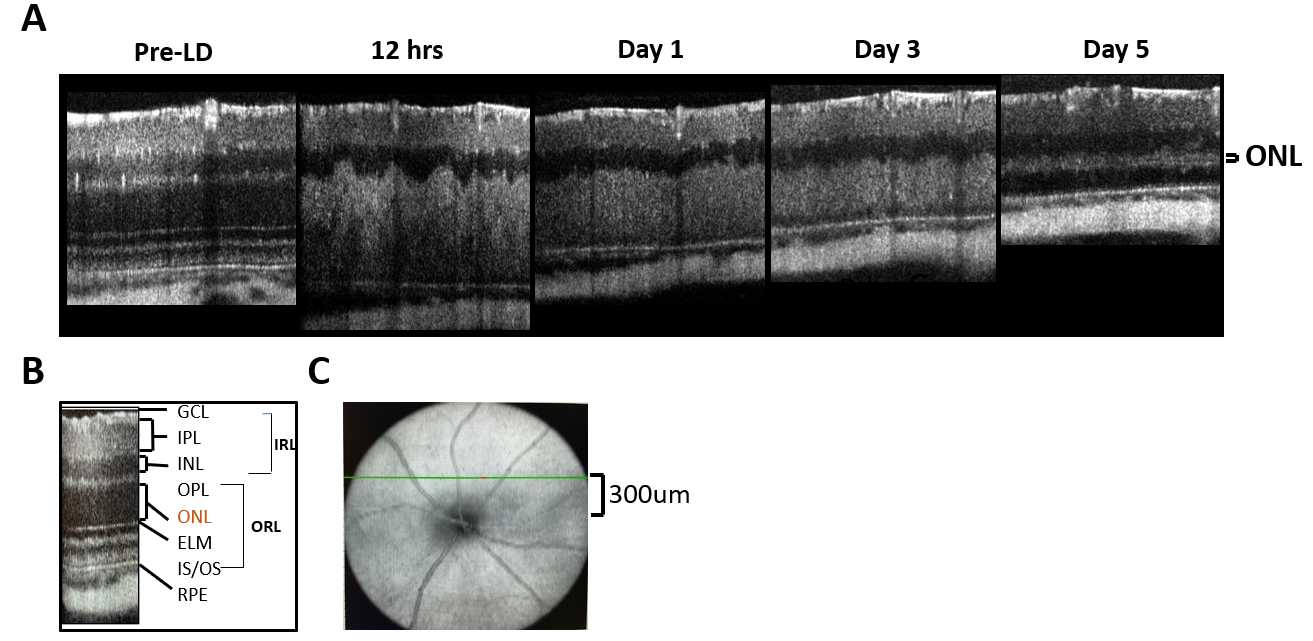


**Figure S1. Post-LD OCT changes of retinas in light exposed mice.** **A** Time course of retina OCT images post light exposure; **B** Representative retinal layers; **C** Position of SD-OCT measurements. Abbreviations: GCL, Ganglion cell layer; IPL, inner plexiform layer; INL, inner nuclear layer; OPL, outer plexiform layer; ONL, outer nuclear layer; ELM, external limiting membrane; IS/OS, inner/outer segment of the photoreceptors; RPE, retinal pigment epithelial.


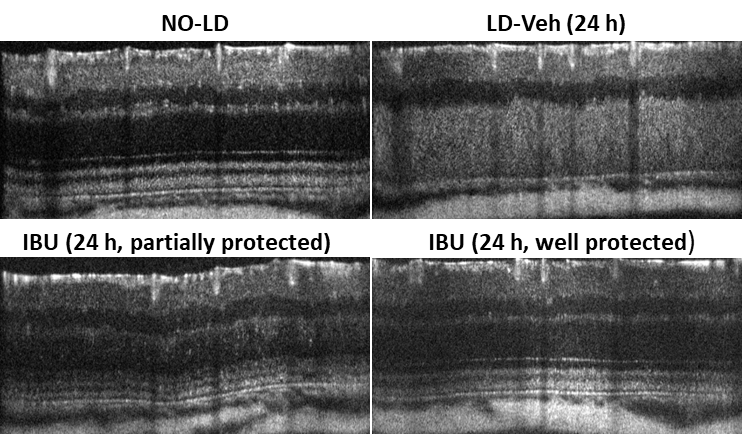


**Figure S2. Representative OCT images of the retina 24 hours post-LD.** The NO-LD group shows normal retinal structure. Vehicle-treated retina displays damage, while IBU-treated retinas show partially to well-protected retinal integrity.


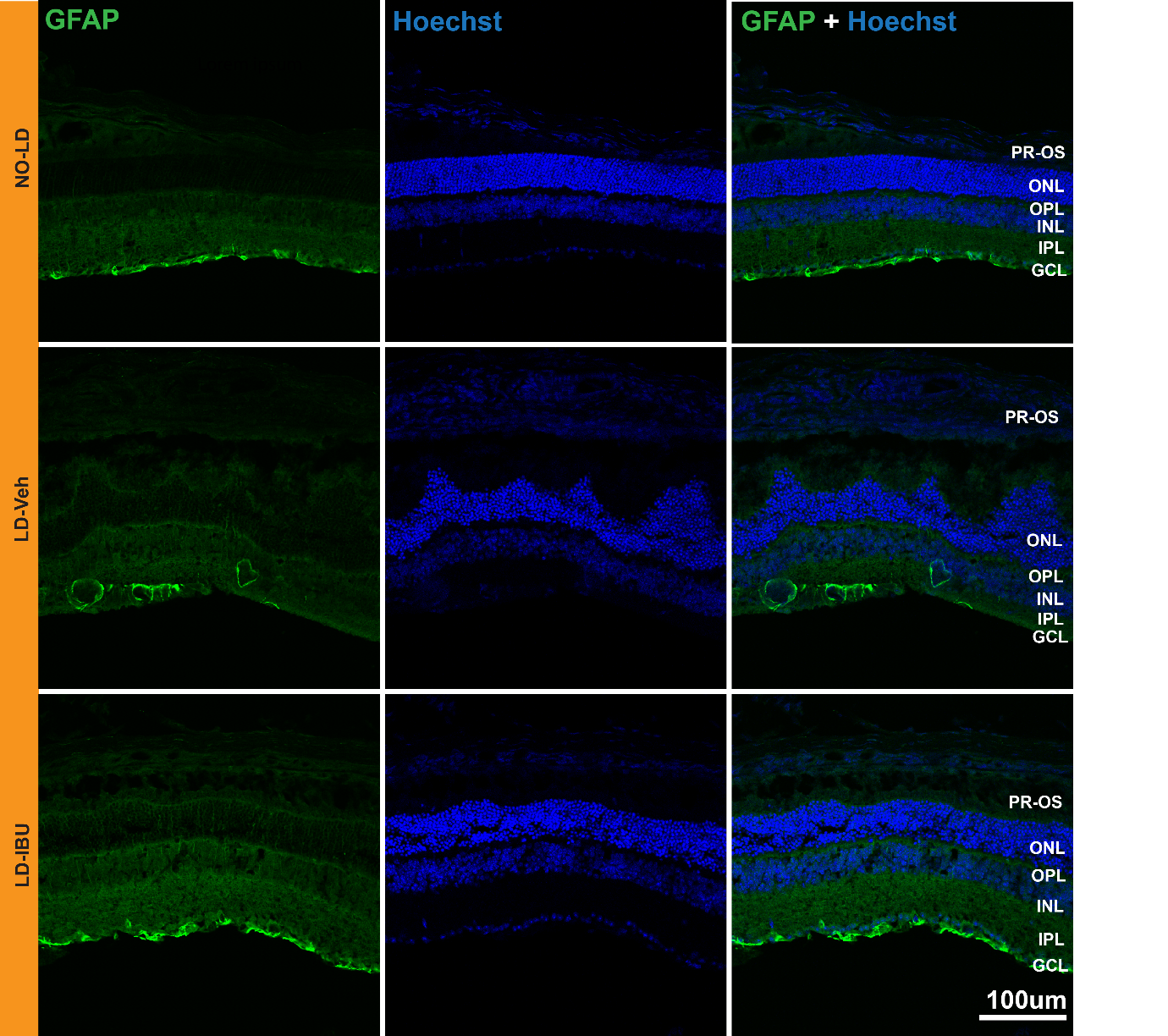


**Figure S3. Comparison of GFAP expression 12 h post LD among three mice groups: No light damage (No-LD), light damage Vehicle (LD-Veh) and light damage IBU (LD-IBU).** 200 mg/kg ibuprofen was used for IP injection. Abbreviations: PR-OS, photoreceptor outer segment; ONL, outer nuclear layer; OPL, Outer plexiform layer; INL, inner nuclear layer; IPL, inner plexiform layer; GCL, ganglion cell layer.


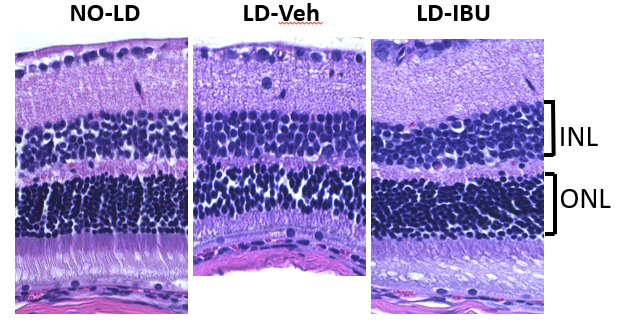


**Figure S4. Histological analysis of retinal sections 72 h post-LD**. The NO-LD group shows intact retinal layers. LD-Vehicle exhibits structural damage, whereas LD-IBU displays preserved retinal architecture, indicating IBU's protective effect. Abbreviations: ONL, outer nuclear layer; INL, inner nuclear layer.


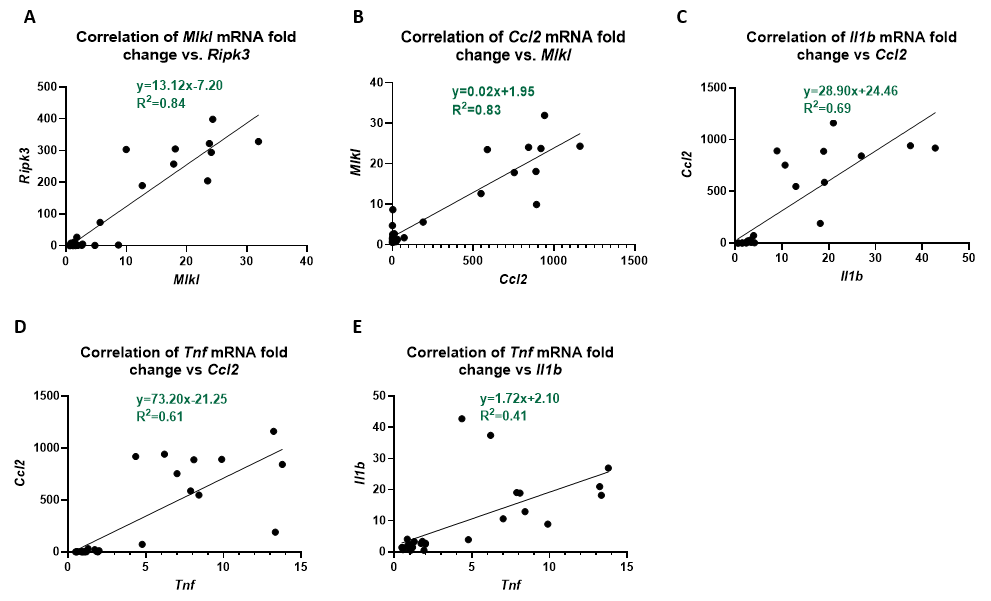


**Figure S5.** **Correlation analysis of mRNA levels of inflammatory markers and *Ripk3*-*Mlkl* pathway 24 h after LD. A** Correlation of *Mlkl* with *Ripk3* mRNA expression (R²=0.78); **B** Correlation of *Ccl2* with *Mkl1* (R²=0.84); **C** Correlation of *Il1b* with *Ccl2* (R²=0.83); **D** Correlation of *Tnf* with *Ccl2* (R²=0.61). **E** Correlation of *Tnf* with *Il1b* (R²=0.41). Linear regression lines and equations are shown on each plot. These relationships indicate significant positive correlations between the expressions of these inflammatory markers and *Ripk3-Mlkl* pathway.
